# Long-Reach DWDM-Passive Optical Fiber Sensor Network for Water Level Monitoring of Spent Fuel Pool in Nuclear Power Plant

**DOI:** 10.3390/s20154218

**Published:** 2020-07-29

**Authors:** Hoon-Keun Lee, Jaeyul Choo, Gangsig Shin, Joonyoung Kim

**Affiliations:** 1 Department of Safety Research, Korea Institute of Nuclear Safety, 62 Gwahak-ro, Yuseong-gu, Daejeon 34142, Korea; hklee@kins.re.kr (H.-K.L.); k534sks@kins.re.kr (G.S.); 2 Department of Instrumentation, Control and Electrical System, Korea Institute of Nuclear Safety, 62 Gwahak-ro, Yuseong-gu, Daejeon 34142, Korea; k728cjy@kins.re.kr; 3 Department of Smart Information Communication Engineering, Sangmyung University, 31 Sangmyungdae-gil, Dongnam-gu, Cheonan-si 31066, Korea

**Keywords:** remote passive sensing, optical fiber sensor network, dense wavelength division multiplexing (DWDM), water level monitoring, spent fuel pool (SFP)

## Abstract

This paper presents a passive optical fiber sensor network based on the dense wavelength division multiplexing (DWDM) to remotely monitor the water level of the spent fuel pool in nuclear power plants. In states of emergency, such as a tsunami, safety information must be secured for rapid response, in spite of all power losses in the plant. We consider the proposed passive sensor network to be one of the best solutions that is able to provide the remote (more than tens of kilometers) monitoring station with the highly reliable on-site information. The principle of water level measurement is based on the change of Fresnel reflection power coefficient at sensing units, which are installed according to the water levels in a row. The sensing units that play the role of reflector and modulator at the same time are connected to an arrayed waveguide grating (AWG) for DWDM. By measuring the spectrum of the optical signal transferred from the sensing units, the water level can be determined in real-time. However, in the remote sensing, the system performance can be seriously degraded due to the Rayleigh Back-Scattering (RBS) of the seeded amplified spontaneous emission (ASE) light that is induced at the fiber-optic link. As such, we investigate the effect of RBS on the remote (more than tens of kilometers) sensing performance of the proposed network. Following the theoretical analysis, we propose a simple network configuration to overcome the RBS issue by utilizing two different transmission paths: one for downstream of the ASE seed light, and the other for upstream of the optical signals coming from the sensing units. Based on the proposed configuration, the maximum sensing distance can be increased up to 42.5 km without the support of any optical amplifier.

## 1. Introduction

In the event of an extreme natural hazard (such as a flood or tsunami) in a nuclear power plant (NPP), it is crucial to provide reliable safety information for the key decision-makers who are responsible for the nuclear safety. This is because it can support a rapid emergency response to the hazardous events through the efficient allocation of the limited resources based on the reliable information. For example, the event at the Fukushima Daiichi NPP demonstrated confusion and misuse of available resources as the instrumentations in the spent fuel pool (SFP) became unavailable [1]. In Korea, when an extreme natural hazard occurs, an emergency operation facility (EOF) is organized, commanding the overall emergency response activities. The EOF site, which has an uninterrupted power supply system as well as an emergency diesel generator, is typically located at least 10 km away from the NPP. Although satellite phones are employed to overcome the loss of data communication between the two distant sites, all power losses at the NPP will lead to the failure of the safety information transfer towards EOF. Thus, it is extremely important to obtain be able to remotely monitor the critical safety parameters such as the SFP water level.

In recent years, a remote sensing network based on fiber-optic sensors has drawn increasing attention thanks to the various advantages in many different fields [2]. For example, it can be applied to various sectors such as hot spot monitoring for electrical power cables [3], security for external intrusion [4], transportation (e.g., railway) monitoring [5], civil infrastructure (e.g., structural health) monitoring [6], and so on. The remote sensing techniques can be classified into two groups according to the spatial distribution of the measurand: (i) distributed sensors and (ii) discrete sensors. The distributed sensors provide continuous monitoring within a specific area/range at relatively high spatial resolutions that were previously realized by Rayleigh-, Brillouin-, or Raman-scattering-based optical time-domain reflectometer (OTDR) [2,6,7]. However, these methods have a trade-off between the spatial resolution and the measurement range [8]. For example, the Raman-based OTDR can provide high spatial resolution (1 cm) for short sensing range (<1 km) [9], or long sensing range (<37 km) at relatively low spatial resolution (17 m) [10]. On the other hand, the discrete level sensors, such as Fabry–Perot sensors or Fiber Bragg Gratings, normally have the purpose of detecting environmental variations (such as temperature, strain, pressure, etc.) at the installed location [11,12,13,14]. Although these sensors can be multiplexed into a single fiber-optic cable so as to provide distributed sensing performance, the number of channels are few [2], limiting the spatial resolution within the target area.

Recently, we proposed and demonstrated a simple all-optical water level monitoring method based on the use of spectrum-sliced amplified spontaneous emission (ASE) light, for its application in nuclear power plant safety [15,16]. This method provides a simple way of multichannel sensing, at a high spatial resolution for low insertion loss, having great robustness to external temperature changes. Although the scheme has great potential for remote sensing as well, its feasibility has not been fully studied. 

In this paper, we investigate the remote sensing characteristics of the DWDM-passive optical fiber sensor network. The proposed sensor network measures the optical power/spectrum of the spectrum-sliced ASE light which is back-reflected at the sensing units (SU) so as to measure the change of Fresnel reflection power coefficient, enabling the water level determination. In this system, we use a small amount of optical power, which is lower than the ASE seed light by 40 dB at the EOF site. It leads the optical signal intensity to be comparable to the Rayleigh Back-Scattering (RBS) of the ASE seed light at the fiber-optic cable, acting as a noise. As such, we investigate the influence of the RBS on the system performances through the theoretical modeling, defining the limiting factors. Following the analysis, we propose a simple network configuration in order to overcome the RBS problem. To be more specific, we separate the transmission paths for the optical downstream and the optical upstream. Consequently, the maximum sensing distance can be increased up to 4 times without using any optical amplification.

## 2. Configuration of DWDM-Passive Optical Fiber Sensor Network

**Figure 1:**
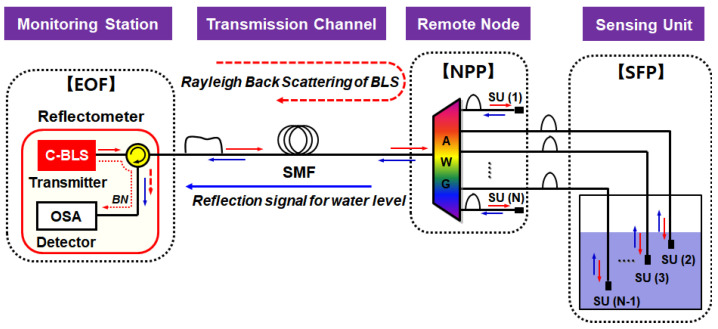
Configuration of the dense wavelength division multiplexing (DWDM)-passive optical fiber sensor network.

The structure of the passive optical fiber sensor network based on DWDM is shown in Figure 1. This architecture can be divided into four function blocks (including one active and three passive blocks): (i) a monitoring station, which includes a reflectometer; (ii) a single mode fiber (SMF) for the transmission channel; (iii) an AWG which is installed at the remote node; and (iv) a sensing unit (SU). The reflectometer, which determines the water level based on the information delivered from the SFP, comprises a Broadband Light Source (BLS) for seeding ASE to the network, an optical spectrum analyzer (OSA) for detecting the reflected signal, and an optical circulator (OC) for separating transmission and detection part. The reflectometer is located at the EOF site as a monitoring station for emergency response. We employ the SMF as a transmission media where its length would be of tens of kilometers. At the equipment room in the NPP (as a remote node), we installed the AWG for (de)multiplexing the ASE lights. The SUs are installed in the SFP where each unit represents a specific water level. Note that two channels of AWG (e.g., the first channel 1 and the last channel N) are used as reference channels that are supposed to be in the air all the time for comparison to other channels [15].

The optical signal path for remote sensing is as follows. First, the C-band BLS at the monitoring station is delivered towards the 1xN AWG of NPP via the optical circulator and the SMF. Then, the transmitted BLS is spectrum-sliced by the AWG according to the dedicated channel wavelengths. The spectrum-sliced ASE lights are delivered to the SUs of SFP. At the end of the SU’s fiber-optic connector, a small portion of the light is back-reflected towards the EOF site, where the reflectance depends on whether the fiber-optic connector is in the air or in the water [15]. The reflected light is multiplexed by the AWG and sent back to the monitoring station via the SMF. Finally, the OSA measures the spectrum of optical signal that contains the water level information in real-time basis.

One of the main drawbacks of long-reach sensing applications is their limited multiplexing capability. Unlike other multiplexing methods, the proposed optical fiber sensor network based on the DWDM technique has very good channel scalability. The channel capacity can be increased easily by utilizing another wavelength band of BLS with the cyclic characteristic of the AWG [17] and/or reducing the channel bandwidth of the AWG [18]. Moreover, it can provide a very simple architecture with off-the-shelf fiber-optic components, and a self-referencing property without requiring previously measured reference information. To compare with other passive remote sensing technologies, we summarized the related state-of-the-art in Table 1.

## 3. Theoretical Model for Remote Sensing

### 3.1. Operational Principle for Water Level Measurement 

The basic principle for water level measurement is based on the Fresnel reflection at the end facet of each sensing unit that is installed in a water pool. The change of surrounding materials (i.e., air or water) at the sensing unit induces a variation of Fresnel reflection power. This is because the Fresnel reflection power coefficient ($R_{m})$ depends on the refractive indices of interfacing materials as represented at Equation (1) [22]:

(1) $$R_{m}={\left(\frac{n_{f}-n_{m}}{n_{f}+n_{m}}\right)}^{2}$$

where $n_{f}$ and $n_{m}$ represent the refractive indices of the optical fiber and surrounding materials (either air or water) at the end facet of the sensing unit, respectively. The sensing unit is a commercial fiber-optic patch-cord including standard SC/PC type connectors, which is based on fluorine-doped silica fiber. In the simulation, we assumed that the refractive index of fiber ($n_{f}$) is 1.4492, air ($n_{air}$) is 1.0002739, and water ($n_{water}$) is 1.3152, respectively [16]. Note that we considered the refractive indices to be constant within the C-band as those are nearly independent of the wavelength and temperature [15]. Based on Equation (1), the power coefficients for the Fresnel reflection are approximately −26.3 dB (0.23% power reflection) in the water ($R_{water}$) and −14.7 dB (3.36% power reflection) in the air ($R_{air}$), respectively. The power difference (*∆P)* between those Fresnel reflections is approximately 11.6 dB, which is well matched with the measurement results [16].

The fiber-optic sensor network exploits the DWDM technique to enable the multichannel sensing capability. For this, we utilize the ASE light (i.e., BLS in Figure 1) and a 32-channel flat-top passband AWG with channel spacing of 100 GHz and 3 dB bandwidth of 80 GHz, subsequently enhancing the measurement resolution. The output light of the BLS has a large bandwidth (>30 nm) where its spectral flatness is less than 2.5 dB [15]. The AWG has relatively low (e.g., <5 dB) insertion loss, which does not visibly vary for wavelengths (i.e., channels). Thus, the intensity of the received optical signal at the reflectometer can be expressed as Equation (2) without taking the RBS effects into consideration [15,16],

(2) $$I_{Signal}\left(\lambda \right)\left[W\right]={\left|E_{ASE}\left(\lambda \right)\right|}^{2}\cdot {\left|\sqrt{T_{AWG}\left(\lambda \right)}\right|}^{4}\cdot R_{m}\cdot 10^{\left(-\frac{L_{IL}}{10}\right)}\cdot \left[1+10^{\left(\frac{BN}{10}\right)}\right]$$

where $E_{ASE}\left(\lambda \right)$ and $T_{AWG}\left(\lambda \right)$ correspond to the electrical field of ASE and the AWG transfer function, respectively. The ASE light can be modeled as uniformly-spaced spectral components that have constant amplitude and uniformly distributed random phase within [0 ~ 2π]. The AWG can be modeled as a series of band-pass filters that have a Gaussian-shape passband [23] as represented in Equation (3):

(3) $$T_{AWG}\left(\lambda \right)=\overset{N}{\underset{i=1}{\sum }}\exp\left[-\ln2{\left(\frac{\lambda -\lambda_{i}}{\Delta \lambda_{BW}/2}\right)}^{2m}\right]$$

where i is the channel number, N is total number of channels, and $\lambda_{i}$ is the center wavelength of each AWG channel where its channel spacing follows the International Telecommunication Union-Telecommunication Standardization Sector (ITU-T) grids. $\Delta \lambda_{BW}$ is the 3 dB bandwidth of each channel, and m represent the filter order for super-Gaussian shape, respectively. The values of simulation parameters for the AWG are as follows, m = 1.415 and $\Delta \lambda_{BW}$ = 0.64 nm, and the channel spacing = 0.8 nm. The insertion loss ($L_{IL}$) in Equation (2) indicates the total optical attenuation throughout the whole signal path, which can be expressed in dB scale as below,

(4) $$L_{IL}\;[\mathrm{dB}]=2\left(L_{OC}+L_{AWG}+L_{SU}+L_{SMF}\right)$$

where $L_{OC}$, $L_{AWG}$, $L_{SU}$, and $L_{SMF}$ represent the insertion losses of optical circulator, AWG, SU, and SMF, respectively. The factor of 2 on the right-hand side means that the system has loop-back structure. The last term of the right-hand side in Equation (2) represents the background noise (BN) of the received optical signal. It specifically means the relative crosstalk of the BLS output at the optical circulator. In the simulation, we select $L_{OC}$, $L_{AWG}$, $L_{SU}$, $L_{SMF}$, and BN to be 1.5 dB, 4.5 dB, 1 dB, 0.2 dB/km, and −56 dB, respectively. These values are based on the previous measurement [16].

### 3.2. The Effect of Rayleigh Back-Scattering Light 

In the fiber-optic sensor network shown in Figure 1, the maximum remote-sensing distance is limited by the Rayleigh Back-Scattering (RBS) of the seeded ASE light at the SMF. The RBS at 1550 nm can be modeled as a function of the SMF length (D) as follows [24,25].

(5) $$R_{RBS}\left[dB\right]=\{\begin{array}{ll} -32-10\cdot \log_{10}\left(\frac{20}{D}\right), & D<20\;\mathrm{km}\\ -32, & D\geq 20\;\mathrm{km} \end{array}$$

As the transmission length (D) increases, the RBS coefficient ($R_{RBS}$) increases also. However, $R_{RBS}$ is saturated to −32 dB when D is >20 km [25]. The power of the received optical signal (at the EOF) is considerably small as compared to that of the seeded ASE light, e.g., <−40 dB when the sensing unit is in the water, leading the RBS effects to be significant. Based on the Equation (5), the intensity of the RBS at the EOF ($I_{RBS}$) can be modeled as

(6) $$I_{RBS}\left(\lambda \right)\left[W\right]={\left|E_{ASE}\left(\lambda \right)\right|}^{2}\cdot 10^{\left(\frac{-2L_{OC}}{10}\right)}\cdot 10^{\left(\frac{R_{RBS}}{10}\right)}.$$

Finally, the total received optical spectrum at the remote station can be obtained as follows.

(7) $$I_{Remote}\left(\lambda \right)\left[W\right]=I_{Signal}\left(\lambda \right)+I_{RBS}\left(\lambda \right).$$

## 4. Analysis Results

### 4.1. Multichannel Sensing Capability with DWDM

**Figure 2:**
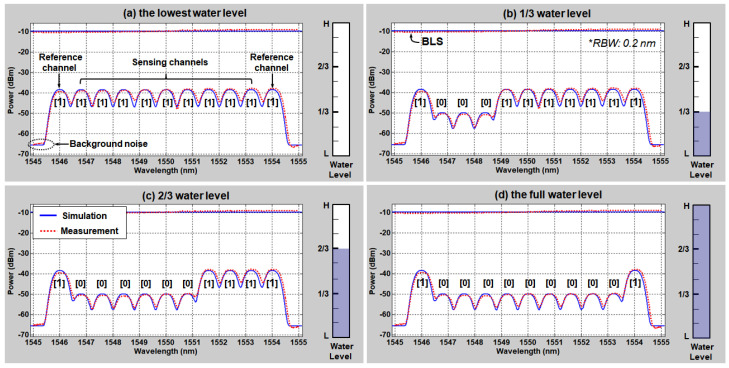
Simulated and measured optical spectra according to the water level at the back-to-back condition (**a**) the lowest water level, (**b**) 1/3 water level, (**c**) 2/3 water level, and (**d**) the full water level.

In this chapter, we characterize the passive optical fiber sensor network using the theoretical model (derived in Chapter 3) that we verified based on the previous experimental demonstration in the back-to-back condition [15,16]. In line with the previous demonstration, we use the 10 nm bandwidth (1545–1555 nm) of the ASE light that corresponds to more than 11 channels of AWG. The output power of BLS was approximately −9.7 dBm/0.2 nm. At the remote node, we used the flat-top passband AWG with 100 GHz channel spacing (transmission bandwidth: 80 GHz or 0.64 nm), which is commercially available for DWDM fiber-optic network applications. The sensing units were connected with the 11 channels of AWG, each port being fiberized with a 2 m optical patch-cord. The sensing units are immersed in a tank filled with water where each unit is attached at designated position. The height of the water tank was ~30 cm and the length for the SMF between the optical circulator and AWG was less than 100 cm. Figure 2 shows the measured and simulated optical spectra of 11 DWDM channels at the (a) lowest water level, (b) 1/3 water level, (c) 2/3 water level, and (d) the full water level, respectively. In addition, the measured and simulated BLS output spectra in front of the optical circulator are plotted in Figure 2a–d. The simulation results show a good agreement with the measured spectra. As shown in the Figure 2, we use 9 channels (channel 2~10) for water-level sensing and 2 channels (channel 1, 11), which are supposed to be in the air all the time, to inform the monitoring station of the reference power level (one is primary and the other is back-up). Regarding the water-level determination process, the measured optical spectrum can be considered to be the amplitude modulated digital signals (that has either “0” or “1” level) with extinction ratio (∆*P*) of 11.6 dB from the perspective of digital optical communication. Thus, if we regard “0” level as the “water” and “1” level as the “air”, it will be possible to use 10 digit patterns for presenting 10 different water levels (e.g., [1 111 111 111 1] means the tank is empty, and [1 000 000 000 1] indicates the tank is full of water). 

### 4.2. Rayleigh Back-Scattering Effects on Remote Sensing Performance 

**Figure 3:**
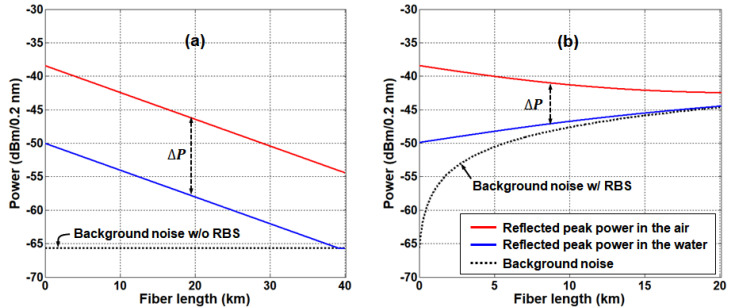
Peak power evolution of each received optical signal reflected in the air and water according to the transmission length (**a**) without the RBS effect (**b**) with the RBS effect.

To investigate the effect of RBS on this sensor network, we analyzed the system performance according to the transmission length by taking the RBS effect into consideration. First, we calculate the power of optical signal at channel 6 (i.e., $I_{Signal}$ at 1550 nm in Equation (2)) as a function of SMF length with no RBS effect included yet. When the SMF induces 0.2 dB/km attenuation, $I_{Signal}\left(\lambda \right)$ for the air and water is shown in Figure 3a, the signal power decreases by 0.4 dB/km as the transmission length increases, while the extinction ratio (∆*P*) was still maintained at 11.6 dB. The background noise power is also plotted in Figure 3a, see the black dashed line. The background noise is mainly attributed to the crosstalk of BLS output at the optical circulator, thus being independent of the fiber length. As shown in Figure 2, the background power was measured to be lower than the BLS output power by 56 dB (i.e., −65.7 dBm/0.2 nm). It should be noted that the AWG channels, apart from the 11 channels in use, were terminated with optical terminators to prevent the unwanted reflection of the ASE lights.

Next, we calculate the RBS power (i.e., $I_{RBS}$ at 1550 nm in Equation (6)) as a function of the transmission length. Then, we add it to $I_{Signal}$ (as in Equation (7)) in order to characterize the RBS effect on the optical sensor network, see Figure 3b. Here, the background noise power increases with the SMF length due to the Rayleigh backscattering light. In spite of the SMF-induced optical attenuation, the Rayleigh backscattering light causes an increase in total received optical power at the monitoring station (see the blue line of Figure 3b), indicating the system performance degradation (i.e., decrease of Δ𝑃).

**Figure 4:**
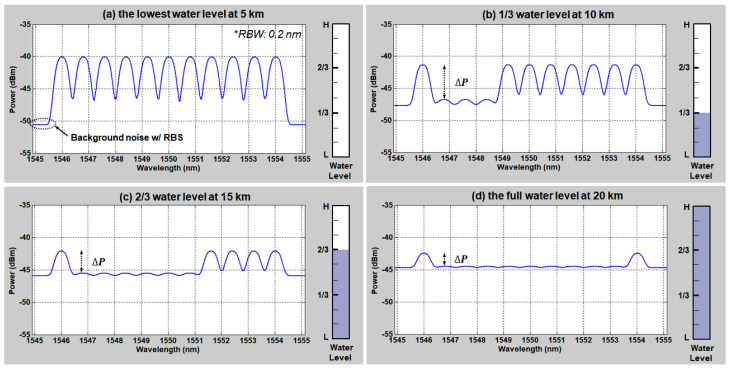
Simulated optical spectra according to the water levels at the different distances with RBS (**a**) the lowest water level at 5 km, (**b**) 1/3 water level at 10 km, (**c**) 2/3 water level at 15 km, and (**d**) the full water level at 20 km.

In addition, we simulated the received optical spectra at several water level conditions and different distances. Four simulation results are shown in Figure 4a–d. Each spectrum represents the remote sensing result of (a) the lowest water level at 5 km, (b) 1/3 water level at 10 km, (c) 2/3 water level at 15 km, and (d) the fullest water level at 20 km distance, respectively. These simulation results are well matched with the calculation results of Figure 3b. In line with Figure 3, it shows increase of the “0” level peak power (i.e., decrease of the extinction ratio (Δ𝑃)) due to the RBS as the transmission distance increases. The “0” level becomes comparable to the background noise power at >10 km SMF length, making it difficult to identify whether the sensing unit is in the water or not. Thus, the maximum distance for this sensor network is limited to 10 km. However, after transmission of 10 km the extinction ratio (Δ𝑃) is still >5 dB, being the minimum margin required to guarantee the correct water level determination regardless of the internal and external environmental changes as previously discussed in [15]. Here, the radiation induced attenuation loss can be also included in the required minimum margin within the additional power margin of 1 dB [26,27].

### 4.3. Mitigation Method for Rayleigh Back-Scattering

**Figure 5:**
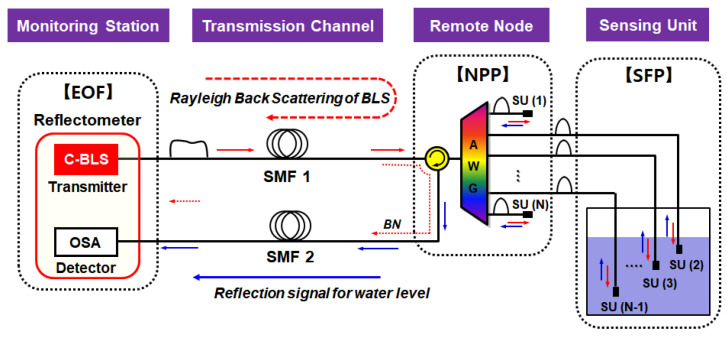
Configuration of proposed network for mitigation of Rayleigh back-scattering (RBS) effect.

Although the proposed DWDM-passive optical fiber sensor network has the advantage of simplicity, its remote sensing capability is limited by the unwanted Rayleigh scattering of the BLS. To mitigate this RBS effect on the sensor network, we propose a simple network configuration that utilizes two different transmission paths as seen in Figure 5.

**Figure 6:**
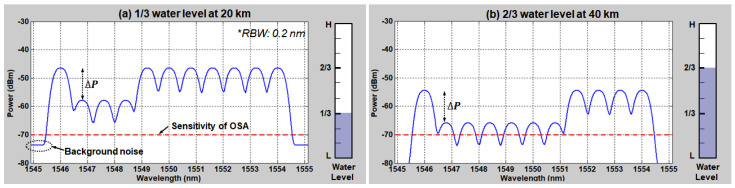
Simulated optical spectra according to the water levels at the different distances with the proposed network configuration: (**a**) 1/3 water level at 20 km and (**b**) 2/3 water level at 40 km.

To be more specific, we separate two transmission paths: SMF1 for transmission of BLS and SMF2 for optical signal. For this, the optical circulator is relocated from the monitoring station (EOF) to the remote node (NPP) at which the AWG is installed. Thus, the power–distance curve will follow Figure 3a rather than Figure 3b. On top of this, background noise (due to the crosstalk at the optical circulator) will experience further SMF-induced attenuation due to the relocation of the optical circulator to NPP. For example, the background noise power is estimated to be −64 dB of the BLS output (i.e., −73.7 dBm/0.2 nm) including fiber loss of 8 dB (20 km × 0.2 dB/km × 2 times) at 20 km distance. Figure 6 shows the simulated optical spectra based on the proposed network configuration. Each optical spectrum represents the transmission result of the 1/3 water level at 20 km and 2/3 water level at 40 km, respectively. As shown in this figure, the transmission length for remote sensing is further increased while still maintaining the peak power differences (∆*P*) of 11.6 dB. However, in this configuration, the maximum achievable length is limited by the detector (OSA) performance. In general, the measured optical signal is supposed to have higher power than the equipment sensitivity to guarantee the reliable instrumentation. Particularly, the sensitivity is directly coupled to the video bandwidth (VBW) and sweep time of the wavelength-scanning instrument. To enhance the sensitivity, the VBW needs to decrease, resulting in a longer sweep time that makes the real-time measurement unavailable, and vice versa. In this analysis, the sensitivity of OSA was set to −70 dBm (RBW: 0.2 nm, VBW: 2.1 KHz), where its typical sweep time is less than 200 ms. Thus, if we consider 3 dB power difference between the reflected peak power from the water (‘0’ level) and the instrument’s sensitivity, the achievable transmission length becomes 42.5 km. It should be noted that the transmission length can be increased further with a high output power BLS or low-loss passive optical components.

## 5. Conclusions and Discussion

This paper presented a remote passive optical fiber sensor network to monitor the water level of spent fuel pool in a nuclear power plant, under the scenario of all power losses in the nuclear power plant caused by extreme natural hazard such as tsunami. In particular, we investigated the remote sensing characteristic of the DWDM-passive optical fiber sensor network that had the multichannel sensing capability. First, the multichannel sensing capability was investigated for the measurement of water level at the back-to-back condition with a simulation and experiment. To increase the spatial resolution, we employed the DWDM method with using a C-band BLS (based on ASE) as a seed light and an AWG for channel multiplexing/de-multiplexing. Then, the system performances were analyzed based on the theoretical model as function of the transmission distance, defining the limiting factor of system. In theory, the maximum transmission length was limited by the Rayleigh Back-Scattering of ASE light to 10 km where the signal extinction ratio was 5 dB. Finally, we proposed a simple network configuration change to eliminate the effect of Rayleigh Back-Scattering. By modifying the network to have two separate transmission paths, the maximum sensing distance was increased up to 42.5 km without any support of optical amplifiers. In this configuration, the transmission length can be limited by the sensitivity of the Optical Spectrum Analyzer. From these results, it is expected that this passive optical fiber sensor network can be used as an auxiliary monitoring system for a spent fuel pool under the emergency situation at the remote location.

In spite of the various advantages of the proposed long-reach optical fiber sensor network, the system performance can be degraded due to the floating particles (such as dust), or small particles dissolved in the water, and condensed water (such as mist or steam). This is because the proposed system is a sort of contact sensor, where the SUs may be stained with these particles or condensed water. As for the particles, its impact can be mitigated considerably by the purification system of SFP that consists of floating removal filters and ion exchangers as previously discussed in [15]. Even if the water quality is maintained through the purification system, the SUs would be contaminated by, e.g., pollutants, radiations, etc. The use of a shielding case will help to prevent the SUs’ end-facet from the contamination. The shielding case is supposed to have several small holes for the water to come in and drain out. Nevertheless, the contaminated SUs need to be replaced with a new one, which can be eased by the segmentation method as previously discussed in [16]. In addition, the condensed water (a drop of water) attached on the end facet of SU can be managed with the adjustment of the installation angle of each SU. By installing the SUs obliquely with a certain angle (or in horizontal direction), not in a vertical direction, the drop of water can be easily removed due to the decrease of surface tensile force of the water drop.

## Figures and Tables

**Table 1:** Comparison of the various passive optical fiber sensor networks based on Broadband Light Source (BLS).

	TDM-OTDR ^1^	TDM-OCDR ^2^	Coarse WDM	Dense WDM
Optical Source	BLS	BLS	BLS	BLS
Interrogator	Photo detector + Oscilloscope	Photo detector + Oscilloscope	Photo detector + DAS ^4^	OSA
Type of SU	Bend-tie sensor	FC type optical fiber connector	FBG	Optical patch cord
Number of channels (including reference channels)	4	10	3	11
Transmission length (km)	50	12	11	42.5
Measurand	Displacement	Water level	Tilt angle, Temperature	Water level
Signal processing	N/A	Required	Required	N/A
Self-referencing information	Required	Provided	Provided	Provided
Remarks	DCF ^3^ is required Simple	Special SU is required Rather complex	Multi-parameter Complex	Channel scalability Very simple
Reference	[19]	[20]	[21]	Proposed method

^1^ TDM-OCDR: TDM-Optical Time Domain Reflectometry, ^2^ TDM-OCDR: TDM-Optical Coherence Domain Reflectometry, ^3^ DCF: Dispersion Compensation Fiber. ^4^ DAS: Data Acquisition System
